# Group level and individual activity of broiler chickens hatched in 3 different systems

**DOI:** 10.1016/j.psj.2023.102706

**Published:** 2023-04-09

**Authors:** Mona F. Giersberg, Roos Molenaar, Ingrid C. de Jong, Kris De Baere, Bas Kemp, Henry van den Brand, T. Bas Rodenburg

**Affiliations:** ⁎ Animals in Science and Society, Department of Population Health Sciences, Faculty of Veterinary Medicine, Utrecht University, PO Box 80163, 3508 TD Utrecht, The Netherlands; † Adaptation Physiology Group, Wageningen University & Research, PO Box 338, 6700 AH Wageningen, The Netherlands; ‡ Wageningen Livestock Research, Wageningen University & Research, PO Box 338, 6700 AH Wageningen, The Netherlands; § Experimental Poultry Centre, Province of Antwerp, Geel 2440, Belgium

**Keywords:** activity, broiler, early feeding, on-farm hatching, tracking

## Abstract

Information on the behavior of chickens hatched in different systems is limited and inconsistent across different studies. Changes in broiler activity can be measured automatically and continuously. The aim of this study was to assess the effects of 3 hatching systems on flock activity using a commercial tracking system, and to compare these findings to individual activity measured under experimental conditions. As this experiment was part of a larger study, it was possible to investigate the effects of vaccination on individual activity. In study 1, flock activity was measured in chickens that hatched either conventionally in the hatchery (**HH**), in a system which provided nutrition in the hatcher (**HF**), or on-farm (**OH**). Chickens were reared in 2 batches, in 12 pens/batch (1,155 animals/pen). One camera recorded top-view images of each pen. A daily activity index (moved pixels/total pixels × 100) was calculated by automated image analysis. In study 2, individual activity was measured under experimental conditions using an ultra-wideband (**UWB**) system. Chickens from the 3 hatching systems were reared in 3 pens (1 pen/treatment, 30 animals/pen). At d14, UWB-tags were attached to 5 chickens/pen, which tracked the distances moved (**DM**). In study 1, group level activity showed a significant age × hatching system interaction (F_8,752_= 5.83, *P* < 0.001). HH and HF chickens showed higher activity levels than OH chickens in wk 1, 4, and 5. In wk 3, higher activity levels were measured in HH compared to HF, and in HF compared to OH pens. In contrast, HH chickens in small groups in study 2 showed lower DM than HF and OH chickens in wk 3 (*P* < 0.001). DM did not differ between treatments before vaccination, however, thereafter, HH chickens showed longer DM, whereas HF and OH chickens moved less. The results indicate that hatching system affected broiler activity at specific ages. Effects found at flock level could not be reproduced by individual measurements in study 2, although stocking density was comparable.

## INTRODUCTION

Hatching in a commercial hatchery involves several procedures which may act as stressors for the chickens at the moment of processing (Giersberg et al., 2020a), but also may have negative effects in the long-term (Hedlund et al., 2021). In addition, the chickens usually do not have access to feed and water until placement at the farm. Though chickens can survive up to 72 h of life without feed and water (van der Wagt et al., 2020), it has also been shown that fasting periods of about 48 h lead to higher mortality rates in broiler chickens at 6 wk of age compared to no fasting and 24 h of fasting (De Jong et al., 2017). However, in the original papers used for this review (van der Wagt et al., 2020) and meta-analysis (De Jong et al., 2017), it was not always indicated clearly which exact age of the chickens (e.g., after emergence from the egg, after pulling or after holding) was regarded as start of the fasting period.

To overcome these potential negative effects, several alternative hatching systems have been developed, which provide early access to feed and water, and involve less intensive handling and processing procedures. One option is to supply early nutrition in special hatching systems in the hatchery (Van der Pol et al., 2015). These systems provide feed, water and continuous light in the hatcher starting after the transfer of eggs to hatcher baskets at about d18 of incubation. After hatch, chickens are minimally handled: they stay in their hatching baskets during processing and transport to the farm. Another alternative is to hatch chickens on-farm (de Jong et al., 2019). In these systems, eggs at transfer time of about d18 of incubation, instead of day-old chickens, are transported to the broiler house. After hatch, the chickens have immediate access to feed, water and light, which are provided in the barn.

Recently, these alternative hatching systems have been compared to conventional hatchery hatching in terms of production performance, animal behavior and welfare. Effects of hatching system on performance seem to be inconsistent (e.g., De Jong et al., 2019; de Jong et al., 2020; Jessen et al., 2021a, b; Souza da Silva et al., 2021). Similar inconsistencies seem to apply to findings regarding behavior and several welfare indicators. In a study comparing traditionally hatchery-hatched (**HH**) and on-farm hatched (**OH**) broilers in a semi-commercial setting, HH chickens responded more actively and less fearfully than OH chickens in several fear-tests (Giersberg et al., 2020b). In contrast, organic OH broilers showed reduced fear of humans and a tendency of less general fear in a novel object test than HH chickens (Jessen et al., 2021b). In a comparison of HH, hatchery-fed (**HF**) and OH chickens, Giersberg et al. (2021) did not find any effects of hatching system on fearfulness. The only welfare indicator more consistently affected by hatching system seems to be footpad dermatitis, with HH chickens scoring worse than chickens from the alternative systems (de Jong et al., 2019, 2020; Giersberg et al., 2021). However, this result could not be reproduced in slower growing broiler chickens in organic housing (Jessen et al., 2021a).

The challenge of these welfare indicators, and particularly of the behavioral observations, is that they only capture few specific moments during the rearing period. Continuous behavioral analyses may provide a more consistent picture, but using direct or video-based methods are often too time-consuming and therefore not feasible. Another possibility may be to use smart technologies to record and analyze behaviors automatically and continuously. However, automatic behavior detection using computer vision approaches has mainly been investigated under experimental conditions. It is largely unknown whether algorithms that function well on the small scale would perform as well in on-farm situations, where animals more often obscure each other, which may reduce the accuracy of the detections (Wurtz et al., 2019).

As a first step, it may therefore be useful to focus on a more general behavior indicator, which can be continuously assessed in a robust way and in various environments. For broiler chickens, activity may be such an indicator. Changes in broiler activity seem to be a promising measure for various health, welfare and performance threats. Sickness and lameness, for instance, have been associated with lower locomotor activity in chickens (Johnson et al., 1993; Aydin et al., 2010; van der Sluis et al., 2021). In addition, activity levels of broilers can be measured in various units (e.g., on an ordinal scale, ranging from low to high levels of activity or more detailed as distances moved in m) and with the help of various technologies. Flock level activity can for instance be assessed by systems based on optical flow patterns (Aydin et al., 2010; de Montis et al., 2013), of which the eYeNamic system (Fancom, the Netherlands) is available for use on commercial farms (Peña Fernández et al., 2018; van Hertem et al., 2018). Individual activity patterns in group housed chickens can be measured by wearable sensors based on passive radio frequency identification or ultra-wideband (**UWB**) technology (van der Sluis et al., 2019, 2020).

The aim of this study was to assess the effects of hatching system on broiler flock activity during the entire rearing period by means of a commercially available automatic tracking system. Chickens from 3 hatching systems were compared: HH, HF and OH. To determine whether the activity patterns measured in broiler flocks could be traced back to an individual level, a second experiment was added, in which individual chickens from the 3 hatching systems kept in small groups were tracked by an UWB system. As this second experiment was part of a larger study (Molenaar et al., 2023), it was also possible to investigate the effects of hatching system on activity after of vaccination, which might be a challenge for the chickens.

## MATERIALS AND METHODS

### Study 1: Group Level Activity

***Experimental Setup and Animals*** Study 1 was carried out at the Experimental Poultry Centre in Geel, Belgium using the same setting and chickens as described by Giersberg et al. (2021) and Souza da Silva et al. (2021). Due to malfunction of the activity tracking system, only the first 2 batches of the 3 batches described by Giersberg et al. (2021) and Souza da Silva et al. (2021) could be observed from May to August 2019. The chickens (Ross 308) were kept in 6 separate rooms of a broiler house, which were accessible from a central hallway. Each room contained 2 adjacent floor pens, each measuring 6.0 × 9.4 m and housing 1,155 chickens at d 0. The pens were separated by wire mesh, had separate feeder and drinker lines, and were littered with a 2-3 cm layer of wood shavings (about 1 kg per m^2^) at d 0. Per hatching system and batch, 2 rooms were used and within 1 room, chickens from the same hatching system were housed in the 2 pens. Allocation of treatments to rooms and pens did not change between batches as the system for on-farm hatching (X-Treck, Vencomatic, Eersel, The Netherlands) was installed permanently in 2 rooms. After hatching of the chicks, the system could be lifted to the ceiling, but it could not be moved to another room.

The age of the parent flock used for the first and the second batch was 28 and 29 wk, respectively. Within one batch, chickens from all 3 treatments originated from the same parent flock. All eggs were incubated at a commercial hatchery (Lagerwey, Lunteren, The Netherlands) during the first 18 d. At embryonic day (**E**) 18, the trays were randomly assigned to the HH, HF, or OH treatment. After candling, HH and HF eggs were transferred to hatching baskets and OH eggs were placed in setter trays. HH eggs were put in a conventional hatcher without light, feed and water. When the majority of HH chickens had hatched at d 21 of incubation, they were pulled from the hatcher and subjected to standard commercial procedures on conveyor belts, including selection of second grade chickens. At E18, baskets with HF eggs were placed in a HatchCare hatcher (HatchTech, Veenendaal, The Netherlands), which provided diode lights and open drinking lines on the sidewalls. After hatching, HF chickens fell from the egg trays on top of the baskets onto the bottom of the baskets. The baskets were equipped with feeding throughs on 2 sides and had holes to reach the drinking lines of the hatcher on 1 other side. During selection of second grade chickens at d 21 of incubation and during transport, HF chickens stayed in the baskets where they had access to the remaining feed but not to water. HH and HF chickens were transported separately after 510 h and 516 h of incubation, respectively, whereas trays with OH eggs were transported at E18. For all 3 treatments, transport took place in conditioned trucks and the transport time to the research farm was approximately 2.5 h.

After arrival at the farm, the trays with OH eggs were placed in the X-Treck system. The caretakers of the farm removed all nonhatched eggs and second grade chicks from the OH pens at d21 of incubation. Starting at E18, continuous light, feed and water were provided in the OH pens. At the day of arrival of HH and HF chickens, the light regime was set up with 1D:23L (d 0) and was reduced to 3L:1D:12L:1D:3L:4D (d7 and onward). Three days before slaughter (d 40), the light regime was returned to 23L:1D. Details on the relative humidity, room temperature and rectal temperature of the chickens, and the nutrient composition of the diet fed are described by Souza da Silva et al. (2021). In short, in the OH pens, a relative humidity of 47% and a room temperature of 34°C were maintained from E18 to d 0. Starting from d 0, the room temperature was gradually decreased to 19°C at d 40 in HH, HF and OH pens. Rectal temperature of the chickens ranged from 41.0° to 41.5°C in all treatments during the first week. Measurements were not conducted at a later age. The diet followed a commercial 4-phase feeding program.

*Activity Measurements* To measure group-level activity, each pen was equipped with a camera of the eYeNamic system (Fancom, Panningen, The Netherlands), which took top-view images of the flocks continuously. A customized software package translated these images every 2 min automatically into an activity index (**AI**). The AI was defined as the number of moved pixels in an image frame divided by the number of total pixels in the image frame, multiplied by 100. For more technical detail on the eYeNamic system see for instance de Montis et al. (2013), Peña Fernández et al. (2018), and van Hertem et al. (2018). Recordings by the eYeNamic system were made during the entire light phase, from d 1 to d 35.

Data on the welfare of the birds from this study have been reported and discussed by Giersberg et al. (2021). Results on the technical performance, including body weight, have been published by Souza da Silva et al. (2021). The experiment was approved by the Institutional Animal Care and Use Committee of the Experimental Poultry Centre, Geel, Belgium (license number EC 2019001).

### Study 2: Individual Activity

*Experimental Setup and Animals* Study 2 was carried out under experimental conditions at the research facility of Wageningen University & Research from November to December 2020. The chickens (Ross 308) were kept in a climate controlled room which contained 3 separate floor pens, each measuring 2 m^2^ and housing 30 chickens. The pens had wire mesh walls, contained a manual feed hopper, 7 nipple drinkers and a metal perch. Birds were fed a standard commercial diet ad libitum and wood shavings were provided as litter material. The chickens hatched in similar systems as described above. One parent flock (age: 27 wk) was used for all 3 treatments. The eggs were incubated at a commercial hatchery (Lagerwey, Lunteren, The Netherlands) until E18. Similar to the procedures of experiment 1, the eggs were then assigned to the HH, HF, and OH treatment. The HH treatment was carried out as in experiment 1. After 510 h of incubation, HH chickens were transported for approximately 0.5 h to the research facility. HF eggs were transported at E18 for 2 h to another hatchery (Probroed&Sloot, Langenboom, The Netherlands), where they were placed in a HatchCare hatcher as described above. After 516h of incubation, HF chickens were transported for approximal 0.75 h to the research facility. OH eggs were transported at E18 for 0.5 h to the research facility.There, they were placed in a small prototype of the X-Treck system, as the experimental pen was too small for the commercial X-Treck system. An egg tray was located in a wooden frame, 22 cm above the floor, with a plastic belt in between. After hatching, the chickens fell on the belt, from which they could reach the floor, and access feed and water provided in the pen. From E18 onward, continuous light was provided in the room. At d 0, when HH and HF chickens arrived, the light regime was set up with 23L:1D for 3 d and was thereafter reduced to 16L:8D (d 9 onwards). Details on the relative humidity and ambient temperature during incubation and brooding, and the nutrient composition of the diet fed are described by Molenaar et al. (2023). In short, a relative humidity of 50 to 60% and a room temperature of 34°C were maintained from E18 to d 0, when only OH chickens were present in the room. After arrival of the HH and HF chickens, room temperature was gradually reduced from 34°C (d 0) to 20°C (d 38). The relative humidity ranged between 50 and 60% from d 0 to d 7 and between 40 and 70% from d 8 to d 38. The diet followed a commercial 3-phase feeding program.

As study 2 was part of a larger study (Molenaar et al., 2023), all chickens obtained a vaccination with life NCD virus, strain C2 (Nobilis CD2, MSD Animal Health, Boxmeer, The Netherlands) at d 0 and 14. In addition, all chickens received a life IB vaccine virus, serotype Massachusetts, strain Ma5 (Nobilis, IB M-A5, MSD Animal Health, Boxmeer, The Netherlands) at d 28, and this was used to assess the inflammation response between the treatment groups (Molenaar et al., 2023). The vaccine was administered individually with 1 drop in the eye and 1 drop in the nose. At d 38, all chickens were killed humanely.

*Activity Measurements* To measure individual activity levels, the chickens were tracked with an UWB system in their home pens. The experimental room was equipped with a Ubisense UWB system (Ubisense Limited, Cambridge, UK) which consisted of 4 receivers placed close to the ceiling in each corner of the room and active tags (model: UBITAG7022, 3.8 × 3.9 cm, weight: 23.4 g including a 12V battery). By default, each tag sent out 2 signals per second (sampling rate = 2/s). These signals allowed for calculating the position of the tags (x, y, z coordinates) using triangulation of the signal between the receivers (based on time of arrival and angle of arrival of the signal). Based on a prior calibration of the experimental room, a customized software (TrackLab, Noldus Information Technology, Wageningen, The Netherlands) could process the coordinates of the tag locations recorded by the UWB system to output variables, such as distance moved (**DM** in m). At d14, the UWB tags were attached to the backs of 5 chickens/treatment by fitting elastic bands around the bases of their wings. Because of the size of the tags, it was not possible to start individual tracking at an earlier age. After 1d of acclimatization to the tags, the distances the birds moved were tracked until d34 for 4h/d during the light phase in the evening. During these times, the chickens were not disturbed by routine work in the room.

Details on the vaccination challenge, the welfare and the technical performance including body weights of the chickens from this experiment can be found elsewhere (Molenaar et al., 2023). The experiment was approved by the Institutional Animal Care and Use Committee of Wageningen University (protocol approval number 2019.D-0002.002).

### Statistical Analyses

Statistical analyses were performed using the SPSS Statistics software (version 26, IBM, Armonk, NY). All data were visually assessed for normal distribution by creating histograms including the Gaussian distribution curve, and homoscedasticity was tested according to the Levene procedure. Depending on distribution, variable structure and characteristics, data were subjected to the following procedures. Study 1: The target variable was the dimensionless, mean Activity Index (AI) per day and the experimental unit was the pen. First, AI was plotted against day and descriptive statistics were calculated. Data were structured by pen within batch, and day as repeated measures. Generalized linear mixed models with a normal distribution and a log link function consisted of the fixed effects hatching system, age, and the interaction between hatching system and age. Room and batch were added as block effects (Giersberg et al., 2021). Study 2: The target variable was the mean DM (m/h) per day and the experimental unit was the individual chicken. Similar to experiment 1, DM was plotted against day for descriptive statistics. To test for differences in DM among hatching systems, a generalized linear mixed model with a normal distribution and a log link function was built. It included hatching system, age, and the interaction between hatching system and age as fixed effects. Data in the generalized linear mixed model were structured by individual chicken, and day as repeated measures. A repeated measures ANOVA was used to analyze the effect of the vaccination challenge on DM. The mean DM (m/h) over 3 d before the vaccination and the mean DM (m/h) over 3 d after the vaccination served as within-subjects factors. The between-subjects factor was “hatching system.” All post hoc pairwise comparisons were adjusted by Bonferroni correction. Differences between the tested parameters were considered to be significant if *P*-values were <0.05. All data are presented as mean ± SE.

## RESULTS

### Study 1: Group Level Activity

**Figure 1:**
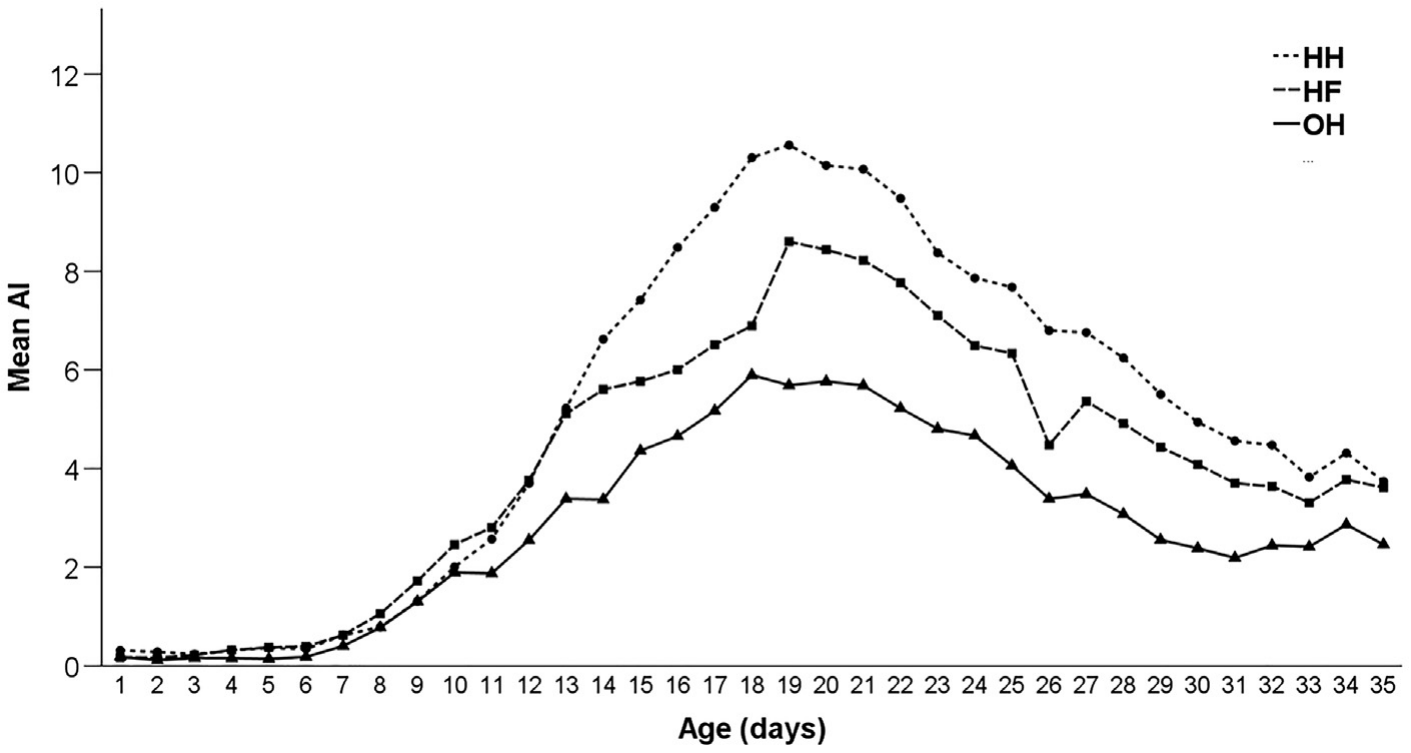
Mean activity index (AI) of hatchery-hatched (HH), hatchery-fed (HF), and on-farm hatched (OH) broiler chickens from d 1 to d 35 of age (study 1). A higher AI indicates a higher level of activity.

*Effects of Hatching System and Age on Group Level Activity* The mean AI over the whole study period (d 0–d 35) was 4.77 ± 0.23 for HH, 4.02 ± 0.19 for HF, and 2.75 ± 0.14 for OH chickens. OH chickens showed a peak of activity (5.90) at d 18, HH (10.56) and HF chickens (8.60) at d 19 (Figure 1). After these peaks, the AI declined in all treatment groups until the end of the experiment

**Figure 2:**
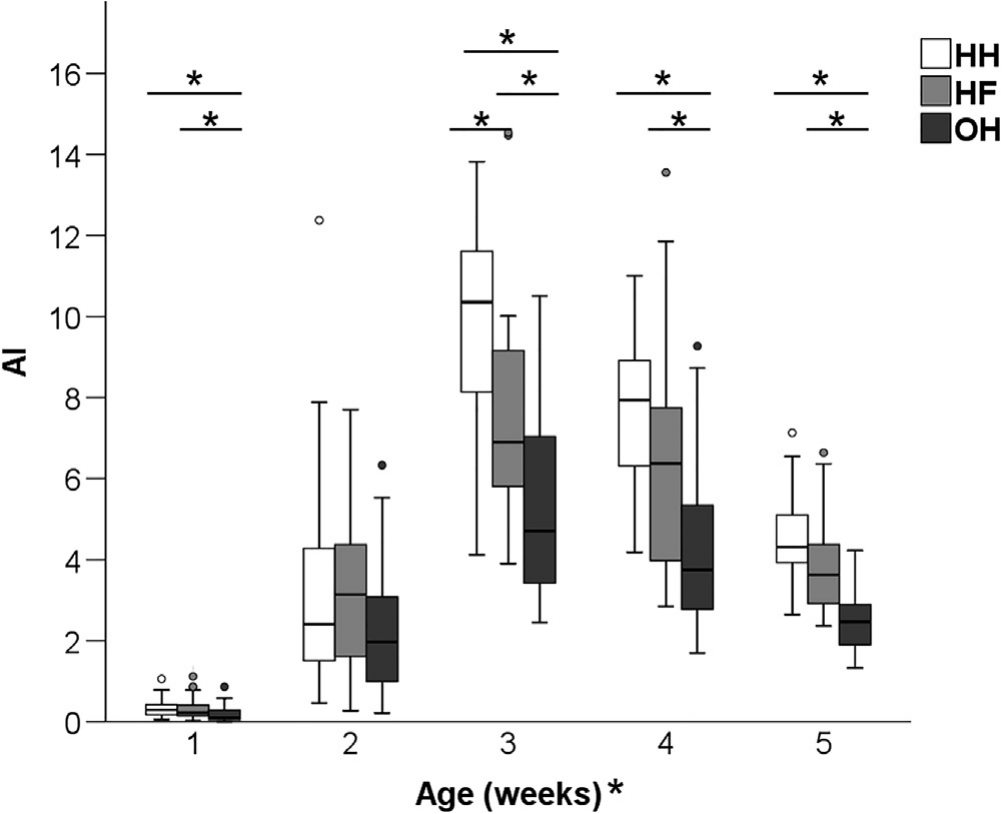
Activity index (AI) of hatchery-hatched (HH), hatchery-fed (HF), and on-farm hatched (OH) broiler chickens at 1, 2, 3, 4, and 5 wk of age (study 1). A higher AI indicates a higher level of activity. * Between bars denotes an effect of hatching system (*P* < 0.05). * After “Age (wk)” denotes an age effect (*P* < 0.05).

Group level activity showed a significant age x hatching system interaction (F_8,752_ = 5.83, *P* < 0.001). Pairwise comparisons showed that HH and HF chickens had a higher AI than OH chickens in wk 1, 4 and 5 (*P* < 0.05) (Figure 2). In wk 3, a higher AI was measured in HH compared to HF pens (*P* < 0.05), and in HF compared to OH pens (*P* < 0.05). In wk 2, activity levels did not differ among hatching systems.

### Study 2: Individual Activity Measurements

**Figure 3:**
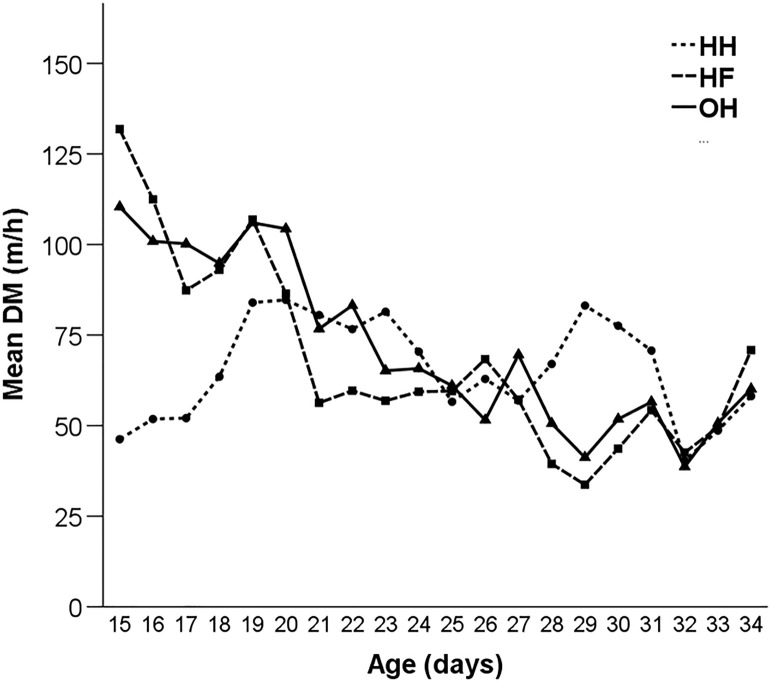
Mean distance moved (DM) (m/h) of hatchery-hatched (HH), hatchery-fed (HF), and on-farm hatched (OH) broiler chickens from d 15 to d 34 of age (study 2).

*Effects of Hatching System and Age on Distances Moved* The mean DM over the whole experimental period (d 15–d 34) was 65.67 ± 2.75 for HH, 68.47 ± 4.11 for HF, and 71.97 ± 3.25 m/h for OH chickens. HF (131.85 m/h) and OH chickens (110.41 m/h) showed a peak in DM at d15 (Figure 3). In HH chickens, 2 peaks in DM were observed: the first at d19 (83.98 m/h) and the second at d29 (83.14 m/h).

**Figure 4:**
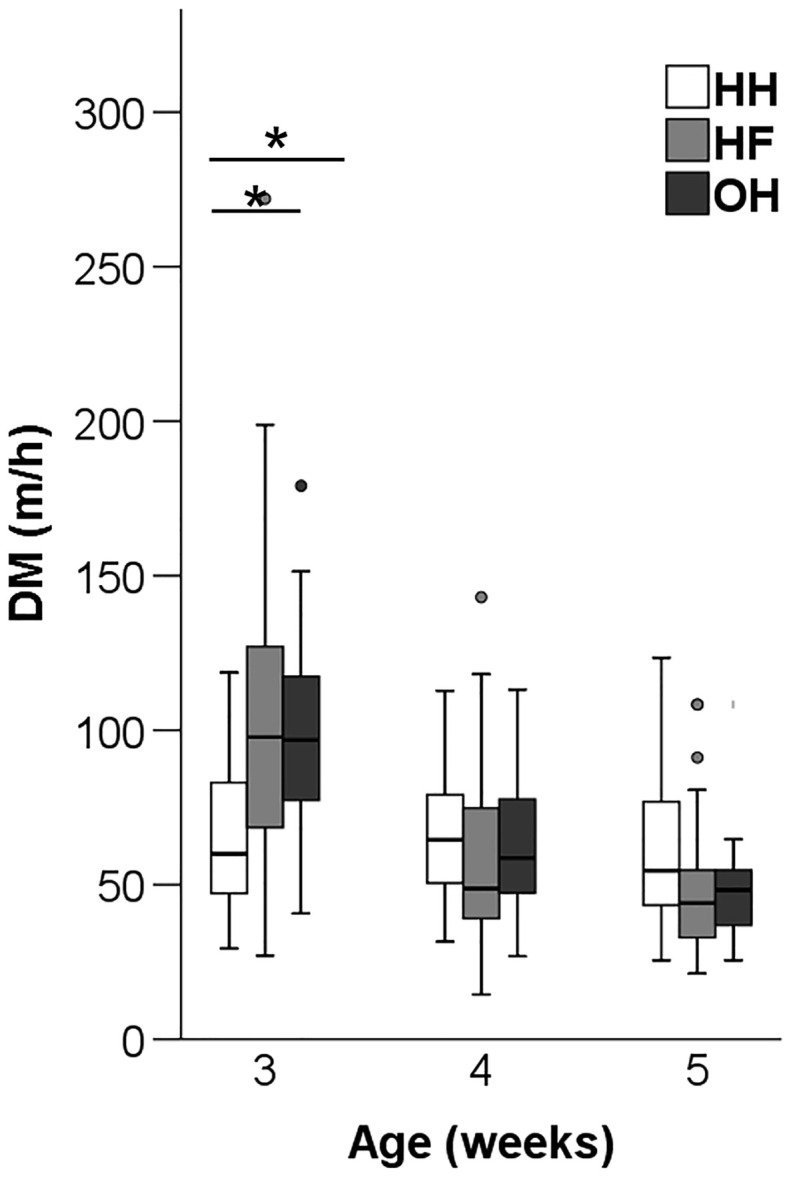
Distance moved (DM) (m/h) of hatchery of hatchery-hatched (HH), hatchery-fed (HF), and on-farm hatched (OH) broiler chickens at 3, 4 and 5 wk of age (study 2). * Between bars denotes an effect of hatching system (*P* < 0.05).

DM measured at an individual level showed a significant age x hatching system interaction (F_4,306_ = 5.57, *P* < 0.001). Pairwise comparisons showed that HH moved shorter distances compared to HF and OH chickens in wk 3 (*P* < 0.001) (Figure 4). In wk 4 and 5, DM did not differ among hatching systems.

**Figure 5:**
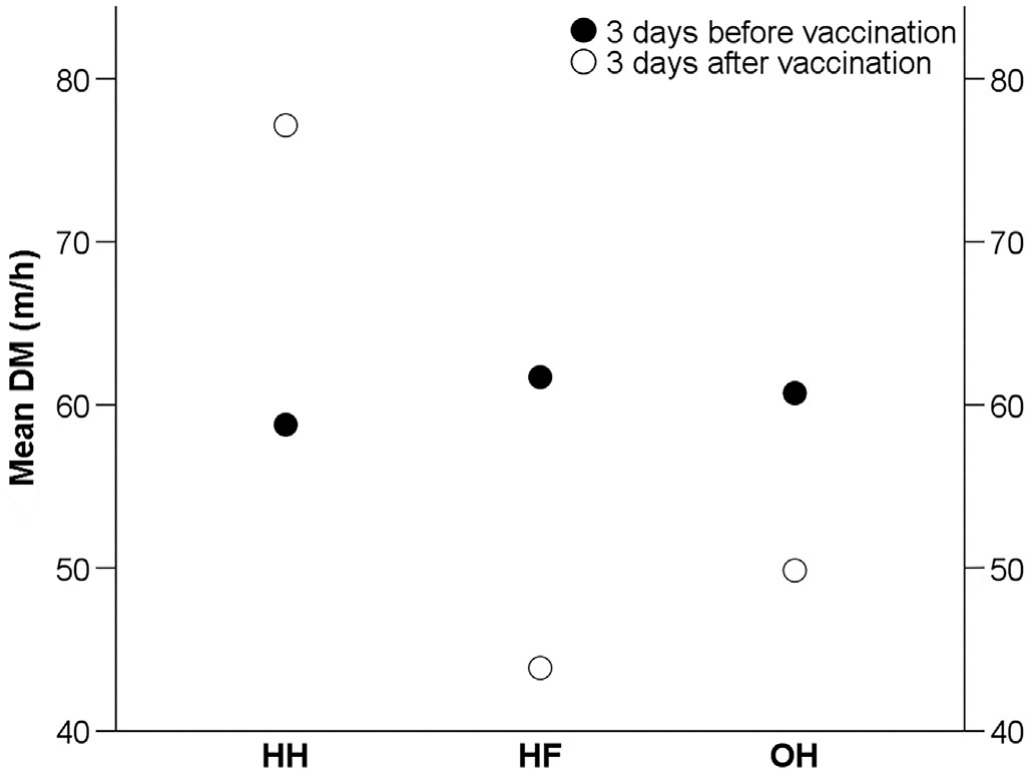
Mean distance moved (DM) (m/h) of hatchery-hatched (HH), hatchery-fed (HF), and on-farm hatched (OH) broiler chickens for 3 d before and 3 d after an IB vaccination at 28 d of age (study 2).

*Effects of Hatching System and Vaccination on Distances Moved* The mean DM over 3 d before the IB vaccination were 58.79 ± 22.17 m/h for HH, 61.70 ± 34.38 for HF, and 60.72 ± 21.42 for OH chickens, and did not differ among hatching system (F_2,20_ = 0.06, *P* = 0.94) (Figure 5). In all groups, the DM of an individual over 3 d before the vaccination differed from its DM over 3 d after the vaccination (F_2,20_ = 8.49, *P* < 0.01). However, the direction of this effect depended on hatching system: HH chickens moved longer distances after the vaccination (77.14 ± 29.38 m/h), whereas HF (43.88 ± 20.47 m/h) and OH chickens (49.85 ± 17.79 m/h) moved shorter distances after the vaccination.

## DISCUSSION

The main objective of the present study was to assess effects of 3 hatching systems on flock activity of broiler chickens during the entire rearing period by means of an automated tracking system. In addition, a small scale experiment was carried out to determine whether or not general flock-level activity could be reproduced by individual activity patterns, and to investigate the effects of a vaccination challenge on broiler activity. In general, activity levels of chickens from all 3 hatching systems were affected by age, showing an increase from post-hatch to approximately 3 wk of age and declining thereafter. Effects of the hatching system on activity were inconsistent and only present at specific ages. Activity patterns detected at the flock-level could not be reproduced with tracking of individual birds.

As with our previous investigations (Giersberg et al., 2021; Souza da Silva et al., 2021), it is important to note that in the current study, both experiments were designed as a system comparison. The 3 different hatching systems, HH, HF, and OH, each consist of several characteristics, which interact and may affect the chickens in various ways. Consequently, it is not possible to relate any effects observed in the chickens to single factors of the hatching environment, such as the presence or absence of transport of day-old chickens or early feeding. The fact that the hatching systems and management procedures described here are available for commercial use underlines the practical relevance of such a system comparison.

### Study 1: Group Level Activity

In general, group level activity declined in chickens from all hatching systems starting in the third week of age (HH and HF after d 19, OH after d 18). This is in line with previous studies in which a drop in time spent walking (Weeks et al., 2000) and distance moved (Van Der Sluis et al., 2019) was found with increasing age of the chickens. This decline in activity may be explained by the decline of walking ability with increasing age, which is often observed in fast-growing broiler chickens (Sørensen et al., 2000; Knowles et al., 2008). However, a decrease in time spent walking with age was also found in broiler chickens with normal gait quality (Weeks et al., 2000). Another factor affecting activity levels of broilers is body weight. It was shown that with increasing body weight and age, locomotion becomes more energetically expensive, which results in adaptative behaviors (e.g., sitting) associated with lower activity levels (Tickle et al., 2018).

The effects of hatching system on group level activity observed for most weeks in the present study remain difficult to explain. During the first week of age, HH chickens may have had a higher motivation to search for potential feed resources in the barn, because they are withheld from feed and water for a certain period, and may thus have been more active. A similar motivation, though to a lesser extent, may have been present in the HF chickens: during transport from the hatchery to the farm, they had access to feed but not to water, and it is questionable whether they actually consumed feed during transport. However, these explanations hardly account for the differences in activity levels among HH, HF and OH chickens observed in wk 3, 4, and 5. By those times, chickens should have been adapted to the barn environment and the locations of resources. The determination of gait scores in the same groups of chickens showed no differences in walking ability among HH, HF and OH chickens (Giersberg et al., 2021). One may argue that the higher levels of activity in HH compared to OH chickens in some weeks may reflect the more active and less fearful responses of HH chickens found earlier (Giersberg et al., 2020b). However, no differences were found among the 3 hatching systems when challenging the chickens of study 1 in behavioral tests (Giersberg et al., 2021). Similar to the effects of age, the effects of hatching system on broiler activity may be explained by differences in body weight. Throughout the study, OH chickens were heavier (about 12%) than HH chickens, with HF chickens having intermediate weights (about 8% heavier than HH chickens) (Souza da Silva et al., 2021). Similarly, van der Sluis et al. (2019) showed that, at the same age, heavier broilers (12-26% heavier) were less active than more lightweight broilers of the same genetic cross.

### Study 2: Individual Activity Measurements

The results of the controlled experiment must be interpretated with caution. Due to the low sample size of pens and tagged birds, the individual chicken was used as experimental unit in the analyses. Although DM were measured individually, it can be questioned to which extent chickens from the same hatching system were independent from one another, as they were housed in the same pen.

Depending on the hatching system, the mean DM recorded in the current study ranged from 65 to 72 m/h. With an average of 19 m/h, van der Sluis et al. (2019) measured lower DM over a similar period of time, in a similar experimental setting and with a similar UWB system. However, van der Sluis et al. (2019) tracked broiler activity from 00:00 to 23:30 h, whereas in the present study, recordings were made during the light phase in the afternoon for 4 h/d. This recording routine was chosen because of limited computing and storage capacity of the UWB system used. It is likely that the lower mean DM measured by van der Sluis et al. (2019) are due to minimal movements of the birds during the dark phase. However, there is no information on the actual hours of light and darkness in their publication (van der Sluis et al., 2019). Activity measured at an individual level declined after a peak in the third week of age in HF and OH chickens, whereas 2 peaks (at d 19 and d 29) were observed in the HH chickens. Similar to study 1, the body weights of the chickens differed among hatching systems. Throughout study 2, HF and OH chickens were heavier (about 8% and 7%, respectively) than HH chickens (Molenaar et al., 2023). It is interesting that the second peak in activity in the HH chickens occurred 1 d after the vaccination challenge. This was also reflected by the more detailed analysis on the days around vaccination: although activity did not differ among chickens from the 3 hatching systems over 3 d before vaccination, HH individuals showed longer DM after the challenge, whereas HF and OH individuals moved less. These lower levels of activity might be due to the chickens’ immune response to the vaccine (Johnson et al., 1993). However, Molenaar et al. (2023) did not find any differences in the humoral immune response (measured as NCD antibody titers) among chickens from the 3 hatching systems. Similarly, pathological changes of the tracheal epithelium, inflammation of the tracheal mucosa after vaccination, and mortality throughout the study did not differ among HH, HF, and OH chickens (Molenaar et al., 2023). Therefore, it remains difficult to explain the higher activity of HH chickens after the vaccination, and it should be investigated whether this effect could be replicated in further studies using a larger sample of birds and pens.

### Relating Group Level to Individual Activity Measurements

It has been shown that age effects on broiler chicken activity are very robust across a variety of housing conditions, study designs and measurement methods (e.g., Weeks et al., 2000; Bokkers and Koene, 2003; Tickle et al., 2018; van der Sluis et al., 2019). However, as shown in the present study, effects of the hatching system on activity levels do not seem to follow similar robust patterns. It was not possible to reproduce the differences in group level activity among the 3 hatching systems when chickens were kept under more controlled conditions and were tracked individually with a different tracking system. The present results show that—in contrast to age effects—potential effects of the hatching system are more vulnerable to differences in environmental conditions among different studies. Therefore, activity, as a more general behavior indicator which can be assessed automatically and continuously, seems to be subject to similar issues as for instance the behavioral tests, which lead to inconsistent results across different studies regarding hatching systems (Giersberg et al., 2020b, 2021; Jessen et al., 2021b). Similar to fear-related responses, the hatching system may affect broiler activity in a more subtle way and it may be also dependent on the birds’ physical and social environment.

Therefore, findings on the effects of certain variables on broiler activity should be interpreted with caution, and generalizations across different studies and measurement methods do not seem possible at the moment. It can be concluded that activity levels, although measured automatically and continuously, seem to be highly dependent on the tracking method used among chickens from the 3 different hatching systems. However, further studies particularly on tracking individual activity of broiler chickens from different hatching systems using a larger number of replicates are necessary.
